# Anchoring effects in the assessment of papers: The proposal for an empirical survey of citing authors

**DOI:** 10.1371/journal.pone.0257307

**Published:** 2021-09-29

**Authors:** Lutz Bornmann, Christian Ganser, Alexander Tekles

**Affiliations:** 1 Science Policy and Strategy Department, Administrative Headquarters of the Max Planck Society, Munich, Germany; 2 Department of Sociology, Ludwig-Maximilians-Universität Munich, Munich, Germany; University of Electronic Science and Technology of China, CHINA

## Abstract

In our planned study, we shall empirically study the assessment of cited papers within the framework of the anchoring-and-adjustment heuristic. We are interested in the question whether citation decisions are (mainly) driven by the quality of cited references. The design of our study is oriented towards the study by Teplitskiy, Duede [10]. We shall undertake a survey of corresponding authors with an available email address in the Web of Science database. The authors are asked to assess the quality of papers that they cited in previous papers. Some authors will be assigned to three treatment groups that receive further information alongside the cited paper: citation information, information on the publishing journal (journal impact factor), or a numerical access code to enter the survey. The control group will not receive any further numerical information. In the statistical analyses, we estimate how (strongly) the quality assessments of the cited papers are adjusted by the respondents to the anchor value (citation, journal, or access code). Thus, we are interested in whether possible adjustments in the assessments can not only be produced by quality-related information (citation or journal), but also by numbers that are not related to quality, i.e. the access code. The results of the study may have important implications for quality assessments of papers by researchers and the role of numbers, citations, and journal metrics in assessment processes.

## 1 Introduction

In a recent study analyzing peer review reports, Hammarfelt, Rushforth [1] identified criteria and strategies that reviewers used to decide on candidates for professorships in Sweden. It is no surprise to read in the paper that bibliometric indicators (especially the popular *h* index) play a prominent role in the assessments of the candidates. The reason for their use is understandable: since no researcher is an expert in all fields within a discipline, it appears reasonable to resort to metrics in cases of fields that are remote from those that are familiar. The use of metrics in situations with great judgment uncertainties may reach its limits, however. Let us suppose a situation in which several candidates for a professorship position are compared. It is the last round of a selection process and the candidates are very similar in terms of their performance. Their previous research papers, funds received and teaching experience have been discussed in great detail. When it comes to the decision regarding the candidates, the summaries for all candidates are provided to the reviewers with the total citation counts. The citation counts are very different, since the candidates are from different fields within economy [see 2].

In thinking about the candidates, one of the reviewers is impressed by the very high citation counts of one candidate (someone from the field of financial economics) and observes that the other candidates are not on the same level. If the reviewer recommends this candidate for the professorship position, the reason for this decision may be the so-called ‘anchoring effect’. Tversky and Kahneman [3] introduced the anchoring-and-adjustment heuristic: “the heuristic maintains that anchoring bias is caused by insufficient adjustment because final judgements are assimilated toward the starting point of a judge’s deliberations … the anchoring effect is the disproportionate influence on decision makers to make judgments that are biased toward an initially presented value” [4]. Research on anchoring effects has shown for various assessment contexts that presented values can have a substantial effect on judgments. Values can even serve as anchors that are not directly related to the assessed objects. Thus, in the example above, it is not only citation counts that could act as an anchor, but also numerical identifiers for the candidates (or any other arbitrary number) presented with the summaries.

In this preregistration, we present the proposal for a study that is intended to investigate various anchoring effects in the assessment of scientific work. To the best of our knowledge, this is the first time that anchoring effects are being investigated in the assessment of scientific work. We found only one study [5] in the area of research evaluation that investigated dependencies of institutional reputation assessments in university rankings on previous rankings within the anchoring framework. The investigation of anchoring effects in research evaluation is an important issue, however. Scientific progress is a key factor for the growing affluence of a society. According to Mormina [6], “the ability to generate scientific and technological knowledge (S&T) and translate it into new products or processes is a key instrument of economic growth and development” [p. 672, see also 7]. A prerequisite for scientific progress is research at the highest possible quality level. Since ‘research quality’ is a normative, ascribing concept [8,9], it depends on assessments by peers (experts in a field). To avoid false quality attributions, the assessments should be unbiased. It inhibit scientific progress if bias factors such as anchoring effects were to significantly affect the assessment.

The design of our study is oriented towards the study by Teplitskiy, Duede [10]. The authors conducted a web-based survey by investigating the referencing behavior of citing authors. Their study had an experimental design, since the authors worked with treatment and control groups and random assignments of respondents to the groups. The respondents assigned to the groups were asked to assess the quality of references that they have cited in publications some years ago. The treatment group differs from the control group in that it receives information on the (true) citation impact of the cited references. The results of the study show that the treatment harms the quality assessments of the cited references.

In our planned study, we shall empirically study the assessment of cited references within the framework of the anchoring-and-adjustment heuristic. Teplitskiy, Duede [10] did not conduct their study within this framework, although the effect of the treatment may be interpreted as an anchoring effect. We shall investigate whether possible adjustments in the assessments of cited references can not only be produced by citation impact information, but also by figures that are not related to quality: are the adjustments specific for citations, or can they be triggered by other figures? An answer on this question may have important implications for quality assessments of papers by researchers and the role of citations in the assessment processes. Quality assessments should be based on research itself, and not on (less relevant) figures. If the assessments are dependent on these figures, their validity may be questioned.

Before we explain our planned research design in more detail below, we present a literature overview of the anchoring-and-adjustment framework (in psychology). This overview is mainly based on two comprehensive overviews published some years ago: Mussweiler, Englich [11]–and the revised chapter published by Bahnik, Englich [12]–and Furnham and Boo [4]. A brief overview of the anchoring effect research can also be found in Kahneman [13].

## 2 Literature overview

Many decisions in research evaluation are judgments that are subject to uncertainty. Although the evaluation tasks are frequently complex and based on ambiguous information, the use of (simple) bibliometric indicators is very popular. Bornmann and Marewski [14] proposed the study of the evaluative use of bibliometrics within the fast-and-frugal heuristics research program. Well selected and informed decision strategies used that are based on bibliometric indicators may serve as heuristics (rules of thumb) in the assessment of scientific work. Bornmann and Marewski [14] argue that such bibliometrics-based heuristics (BBHs) may lead to decisions in certain research evaluation situations that are at least similarly good as expensive peer review processes (that may or may not be informed by indicators). This argument is based on the results of numerous empirical studies (in psychology) that have demonstrated the comparable good performance of heuristics compared with other, more complex approaches using full information in various decision contexts.

From the mid-1970s onwards, research on decision making also revealed another side of the heuristics coin. This research is mainly based on the pioneering work by Tversky and Kahneman [3]. Their study demonstrated that the use of heuristics may lead to biases in decision making processes. The respondents in the study were asked to estimate the percentage of African nations in the UN. An arbitrary number presumably produced by a wheel of fortune was used as a possible anchor in the estimation process: the respondents were requested to estimate whether the percentage is higher or lower than the anchor. A 20 percentage-point difference in the mean estimations (of the subsequent absolute anchoring question) revealed the strong effect of the anchor that was randomly induced. Thus, the results indicate that the estimates were (strongly) adjusted by the respondents to the anchor value.

One might think that the results by Tversky and Kahneman [3] are restricted to judgments without important implications. The results by Englich and Mussweiler [15] revealed that this is not the case, however: experienced trial judges were influenced by sentencing demands, and this influence could also be observed in situations in which the demands came from non-experts. Since the appearance of the study by Tversky and Kahneman [3], many studies have investigated anchoring effects in very different contexts. Descriptive overviews of these studies in tabular form can be found in Furnham and Boo [4]. The literature overviews of Mussweiler, Englich [11] and Furnham and Boo [4] came to very similar conclusions in their overall assessments of the prevalence of anchoring effects [see also 12]: “anchoring effects are among the most robust and easily replicated findings in psychology” [11]. Furnham and Boo [4] concluded as follows: “research in the field demonstrates that anchoring is a pervasive and robust effect in human decisions” (p. 41). Previous research even revealed that anchoring effects appeared in situations in which explicit instructions exist to correct their effect.

The robustness of the anchoring effect can be assumed, since the effect seems to exist independently of many moderating variables. Anchoring effects have been shown in comparative judgment tasks (e.g. is something larger or smaller) and in absolute judgment tasks (e.g. how high, long, or great is something) [11]. The effect seems to be independent of the extremity of the anchor (although it should not be implausibly extreme). Anchors may be numeric values or certain stimuli. Decision tasks in anchoring effect studies were trivial (e.g. an estimate of the percentage of African nations in the UN), or apocalyptic (e.g. the estimated likelihood of a nuclear war). Robust anchoring effects could be observed not only in laboratory settings such as those used by Tversky and Kahneman [3], but also in ‘real-world’ settings such as the legal judgment settings in Englich and Mussweiler [15]. Another typical ‘real-world’ setting in anchoring effect studies is consumer behavior: consumer behavior appears to be influenced by price proposals that serve as anchors.

Studies have differentiated whether or not the anchor should be relevant for the judgmental task in order to be effective. In our study, for example, citations may be interpreted as relevant anchors for the assessment of papers, but arbitrary identifiers of papers can be interpreted as being irrelevant for quality assessments. Although one might expect that only relevant anchors are effective, Furnham and Boo [4] concluded–based on the literature–that “irrelevant anchors produce similar effects in judgmental decisions in comparison to those of informational relevance anchors” (p. 38). Research also revealed that anchoring effects are observable in situations in which participants tried to work against the anchors’ influence or received a prize for presenting the best estimate.

Anchoring is usually used as a descriptive concept by pointing to an assimilation or contrast [16]. Some studies have provided possible mechanisms for explaining the observed anchoring effects in the various contexts. The proposed mechanisms have been summarized by Mussweiler, Englich [11] as follows: “At least three mechanisms may influence the initial stage of standard selection. First, a particular value may be selected as an anchor because conversational inferences suggest it as relevant. If a particular anchor is explicitly mentioned by the experimenter, then judges may well use it to subsequently compare it to the target. Second, a value may be selected as an anchor because it is easily accessible and comes to mind during the evaluation of the target. Finally, an anchor may be self-generated via an insufficient adjustment process. Judges who are provided with an implausible anchor, for example, may use this value as a starting point to generate a more plausible value, which is then compared to the target. This suggests that the alternative mechanisms of conversational inference, numeric priming, and insufficient adjustment may contribute to the selection of an anchor value” (pp. 195–196).

## 3 Study design

The numerous studies on anchoring effects show that the assimilation of judgments occurs in many natural settings (e.g. pricing or legal judgments) and thus has a great practical significance in judgments by experts and non-experts. According to Englich and Mussweiler [15], “anchoring is a pervasive and robust effect in human judgment that reliably influences numeric estimates in a variety of natural settings” (p. 1537). In the planned study, we shall investigate anchoring effects in the assessment of scientific work. We shall undertake a survey of corresponding authors with an available email address in the Web of Science database (a multi-disciplinary literature database including citations). The survey will be similarly designed as the survey conducted by Teplitskiy, Duede [10]. The questionnaire used by the authors can be found in their supplementary information. The Research Ethics Committee of Social Sciences at Ludwig-Maximilians-University (LMU) Munich has reviewed our planned survey regarding ethical aspects of the proposed research. The committee came to the following conclusion: there are no objections to the implementation of the research project.

The corresponding author receives an email with a link to a web-based questionnaire. The author is asked for the assessment of a paper (reference) that he/she cited in a previous publication. The authors are randomly assigned to different experimental settings in which they receive (or not) (1) a randomly generated anchor that is not related to the quality of publications, (2) information about the (true) citation impact of the paper (as an anchor with possibly relevant information), or (3) information about the (true) citation impact of the journal in which the paper was published (as an anchor with possibly relevant information).

Teplitskiy, Duede [10] used the following cover letter in the email of their survey to the authors: “The *Laboratory for Innovation Science at Harvard* would like to invite you to take part in a quick (5–10 minute) survey about how researchers reference existing work when writing papers. Although citations and related metrics like the *h-index* are widely used in academia to evaluate research and allocate resources, the referencing decisions on which they are based are poorly understood. Consequently, we have selected your paper … and want to ask you about two specific references within it. Your input will help us and the broader scientific community assess the validity and limitations of existing ways of evaluating and ranking scientific works and possibly develop superior alternatives.” When the respondents decided to take part and started the survey, they were asked whether they remembered the presented references (or not). One group received additional citation impact information and the other did not. Further questions referred to how well the authors knew the paper (‘extremely well’ through to ‘not well’), how much the reference influenced the research choices in the citing paper, and which aspects of the paper were influenced by the cited references. In the final part of the questionnaire, the respondents rated the reference against possible others in the field concerning several characteristics (quality, novelty, significance, validity, generalizability, and canonical reference).

In the planned study, we shall replicate this survey design by Teplitskiy, Duede [10]. Since the replication will take place within the heuristics framework (see section 2), we shall change the design in several ways:

1. Presentation of a journal metric: Teplitskiy, Duede [10] provided citation counts of the cited publications and its citation percentile in the questionnaire. The citation percentile is the percentage of papers in a subject category (and publication year) that received (equal to or) fewer citations than the focal paper [17]. In the questionnaire, we shall present not only information on the citation percentile of single papers, but also the citation impact of the journal in which the cited reference has been published. The information on the journal impact will also be presented as percentile information: the percentage of journals in a subject category (and publication year) that received (equal to or) fewer citations than the focal journal in terms of the popular journal impact factor. The impact of the journal may also serve as anchor for the respondents in assessing the cited reference. The study by Waltman and Traag [18] indicates that the value of a paper may be interpreted within two perspectives: the number of received citations and the reputation of the publishing journal. In our study, we are interested in whether the two perspectives lead to different anchoring effects in the assessments of the cited references.
2. Randomly generated anchor with irrelevant information: The citation information provided in the survey by Teplitskiy, Duede [10] may be interpreted as an anchor providing relevant information for the assessment of the quality of the cited references. In the planned study, we shall consider another anchor with irrelevant numerical information [see 19]. This second anchor will be presented as an access code in the cover letter (see above). The access code is randomly generated from numbers between 0 and 100. We focus on this range to be in the same range as citation and journal percentiles. The respondents are asked for copying the code in the web-based questionnaire [see here 20]. All respondents who do not receive the anchor stimulus will be issued a sequence of randomly generated letters as an entering code.
3. Assessment of only one cited reference: Respondents in the study by Teplitskiy, Duede [10] were requested to assess more than one cited reference. We shall deviate from this approach: each corresponding author is asked to assess only one cited reference. The presentation of more than one cited reference may lead to undesirable dependencies in the assessment of cited references. Another advantage is the reduction in time for working on the web-based questionnaire.
4. Assessment of the same cited reference by many respondents: We shall consider only papers with references in our study that have been cited by more than one citing corresponding author in our dataset. Each cited reference will be assessed under three treatment group conditions (citation, journal, and access code) and the control group conditions. This enables us to control the effect of the cited reference in the statistical analyses (by using models for repeated measures).

The bibliometric data that we shall use in our study originate from an in-house database developed and maintained by the Max Planck Digital Library (MPDL, Munich) and derived from the Science Citation Index Expanded (SCI-E), Social Sciences Citation Index (SSCI), Arts and Humanities Citation Index (AHCI) prepared by Clarivate Analytics. We shall focus on papers published in 2018 or 2019, since we anticipate better memory performance of the citing authors for recent papers than for papers published many years ago. In the database, there are 5,608,611 papers published in 2018 or 2019. Of these papers, we shall consider only articles, since different types of documents may lead to different selection decisions of cited references by citing authors at that time. For reviews, cited references were selected (and read) to be included in an overview of research; for articles, cited references were more likely to be the primary starting point for own research.

In our study, articles are only considered if they have a single email address for the corresponding author in the database (some papers in the database have more than one email address). In order to identify the corresponding authors to be contacted, we included only papers that have at least one article as a linked cited reference in our database. For a linked cited reference, a corresponding source document exists in the Web of Science database. Further metadata, such as paper title, citation counts, and journal impact factor only exist for linked cited references. Furthermore, we only included those papers with a similar number of cited references, in order to control a possible effect of the number of cited references on how well the citing authors can remember a particular cited reference. For this purpose, we calculated the mode number of cited references based on the papers remaining after the aforementioned restrictions (which is 30 in our dataset). We only kept those papers from this set whose number of cited references does not differ from the mode number of cited references by more than 1.

For each of these papers, we generated the pairs of the email address of the corresponding author and the linked cited references (with the ‘article’ document type) of the papers. The pairs for which both papers share at least one author name are excluded from this set in order to exclude self-citations. For each paper occurring as a cited reference in the resulting pairs, we selected 8 (or, if possible, the largest multiple of 4) email addresses, while at the same time, each email address is selected not more than once. Note that several citing papers may have the same email address. In such cases, a maximum of 1 cited reference is also selected for an email address (instead of selecting 1 cited reference for each citing paper). We used a greedy algorithm [i.e. an algorithm using a heuristic to find a locally optimal choice at each stage of the algorithm; see 21] that aims to select as many pairs of email addresses and cited references that fulfil the aforementioned conditions as possible. This algorithm iteratively searches for 8 email addresses (that have not been selected in previous iterations) for each cited reference, starting with the cited references that have been cited by the fewest email addresses in the data. After iterating over all cited references, this process is repeated–searching for 4 instead of 8 email addresses for each cited reference–until there is no cited reference left for which at least 4 email addresses could be found that have not been selected earlier in the process.

This process results in 36,592 email addresses and 3,611 cited references. Each of the 36,592 corresponding authors is assigned randomly to one of the three treatment groups or the control group. Each treatment group receives one of the three items of information: citation percentile (for the cited article), journal percentile (for the cited article), or access code. The authors in the control group do not receive any of this information. This will result in 9,148 authors for each group. Teplitskiy, Duede [10] sent an email to 63,049 corresponding authors. About 15% reached the last page of their survey. If we have a similar response rate, we shall have a sample of n = 5,489. In our study, the email addresses will only be used for inviting the respondents. After collecting the data, the email addresses are deleted and the anonymized data are analyzed which will not allow the identification of respondents.

In our sample, we shall have corresponding authors with different levels of experience in their field. We anticipate that the assessments of cited references are dependent on this level: senior researchers may be in a better position to assess the quality of a cited reference than junior researchers. There may be the risk, therefore, that junior researchers are more open to biases in their assessments. In our study, we target these differences between the corresponding authors interviewed by means of their random assignments to the treatment and control groups. Furthermore, the literature overview by Furnham and Boo [4] shows that “expertise does not significantly reduce the assimilative bias in decisions that affect inexperienced laypeople” (p. 39).

## 4 Statistical analysis

The web-based survey will yield quality assessments of cited articles (concerning quality, novelty, significance, validity, generalizability, and canonical reference) on a percentile scale from 1 (very poor) to 100 (exceptional) whereby 50 denotes a typical paper. These quality assessments are the dependent variable, and we are interested in how they relate to the independent variables: citation, journal, and access code (in terms of percentile values). Our dataset consists of repeated measures, since each cited reference has been assessed under up to four conditions (three treatments): by presenting citation percentile, journal percentile, access code, or no further information.

Table 1 shows fictitious data from the web-based survey for a cited reference. Cited article 1 received more citation impact than 40% of the articles from the same subject categories and publication year, and was published in a journal with a higher citation impact than 22% of the journals in the same subject category and year. This information was presented to respondents 1 and 2. Respondent 3 entered the survey with the access code 80. Respondent 4 assessed the cited reference without indicating an access code in terms of numbers (but letters) or receiving citation impact information (on the paper or journal level).

**Table 1:** Fictitious data that are paper-based (e.g. the citation percentile) or respondent-based (e.g. the quality assessment).

	Citation	Journal	Access code	No information
**Paper data**				
Percentile	40	22	80	
**Survey data from four respondents**	1	2	3	4
Quality	35	25	45	20
Novelty	30	45	22	35
Significance	21	32	78	74
Validity	34	47	57	76
Generalizability	44	55	83	42
Canonical reference	20	12	15	95

We plan to analyze the data with multilevel models for repeated measures (i.e. at different conditions). We shall estimate one model for each quality assessment of cited articles. In each model, we examine how the various information at a certain condition (e.g. different citation percentiles at the citation impact condition) is related to the quality assessments. Fictitious data for three cited articles are reported in the long format in Table 2.

**Table 2:** Fictitious data in the long format for one quality dimension.

Cited article	Respondent	Condition	Percentile		Quality assessment
			Value	Above 50	
1	1	1	40	0	35
1	2	2	22	0	25
1	3	3	80	1	45
1	4	4			20
2	5	1	61	1	16
2	6	2	15	0	41
2	7	3	22	0	20
2	8	4			20
3	9	1	25	0	11
3	10	2	80	1	61
3	11	3	35	0	36
3	12	4			65

Note. 1 = citation, 2 = journal, 3 = access code, 4 = no information.

In the first step of the analysis, we compare average quality assessments of articles above and below a percentile value of 50 (i.e. excluding papers at the median) for the three treatment and one control groups. Thus, we have seven arithmetic means: citation percentile above/below median, journal percentile above/below median, access code above/below median, and no information. We anticipate that quality assessments will be lower for articles below the median values and higher for articles above the medians.

To control for unobserved characteristics of the cited articles, in the second step of the analysis, we fit regression models with article fixed effects:

$$\begin{array}{l} {\mathrm{quality}\;\mathrm{assessment}}_{ij}=\beta_{0}+\beta_{1}\mathrm{condition}2_{ij}+\beta_{2}\mathrm{condition}3_{ij}+\beta_{3}{\mathrm{abovemedian}}_{ij}\\ \;+\beta_{4}\mathrm{condition}2_{ij}\cdot {\mathrm{abovemedian}}_{ij}+\beta_{5}\mathrm{condition}3_{ij}\cdot {\mathrm{abovemedian}}_{ij}+v_{j}+e_{ij} \end{array}$$

whereby *quality assessment*_*ij*_ is the quality assessment at condition *i* for cited article *j*, *condition2*_*ij*_ and *condition3*_*ij*_ are dummy variables indicating the respective conditions, *abovemedian*_*ij*_ is a dummy indicating whether the cited article is below (= 0) or above (= 1) the median, *v*_*j*_ is the article effect, and *e*_*ij*_ is an idiosyncratic error term.

**Fig 1:**
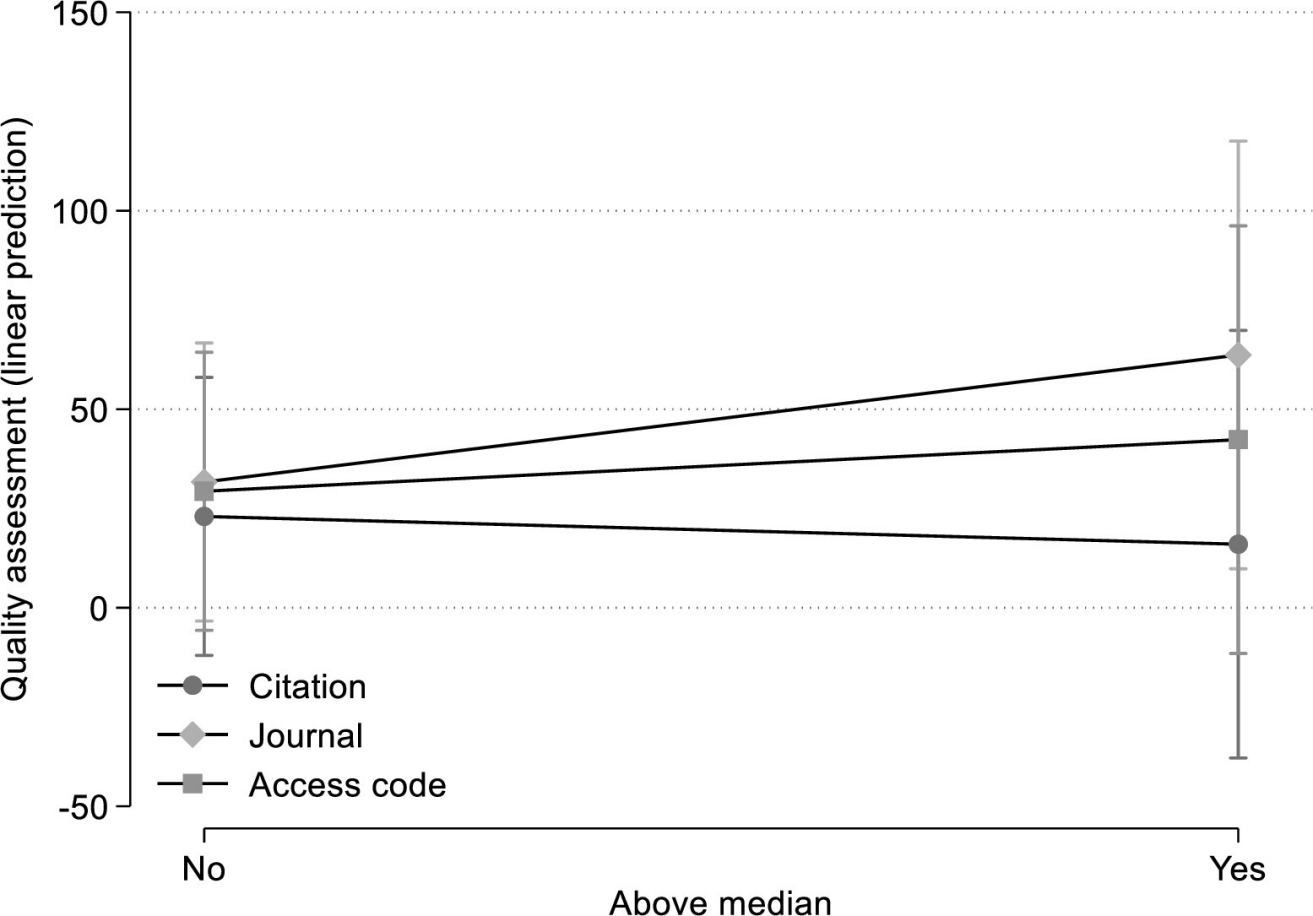
Relationships between treatments (citation, journal, and access code) and quality assessment.

We plan to present the results of the model estimations in terms of predicted values. Using the example data in Table 2, this would yield the results in Fig 1. The results show that for articles with citation and journal impact percentiles below the median as well as for access code ‘percentiles’ below the median, the expected quality assessment is about 25 to 30, controlling for article characteristics. In this fictitious example, higher journal impact factors and access codes have a positive effect on quality assessments, as the expected values are higher for articles above the median (63 and 42, respectively). By contrast, the presentation of citation percentiles in the survey would negatively affect the quality assessments.

In the third step of the analysis, we examine treatment effect heterogeneity by estimating fixed effects regression models, including the percentile values.

The model is specified as follows:

$$\begin{array}{l} \mathrm{quality}\;{\mathrm{assessment}}_{ij}=\beta_{0}+\beta_{1}\mathrm{condition}2_{ij}+\beta_{2}\mathrm{condition}3_{ij}\\ \;+\beta_{3}\mathrm{condition}1\cdot \mathrm{percentile}+\beta_{4}\mathrm{condition}2\cdot \mathrm{percentile}+\beta_{5}\mathrm{condition}3\cdot \mathrm{percentile}\\ \;+v_{j}+e_{ij} \end{array}$$

**Fig 2:**
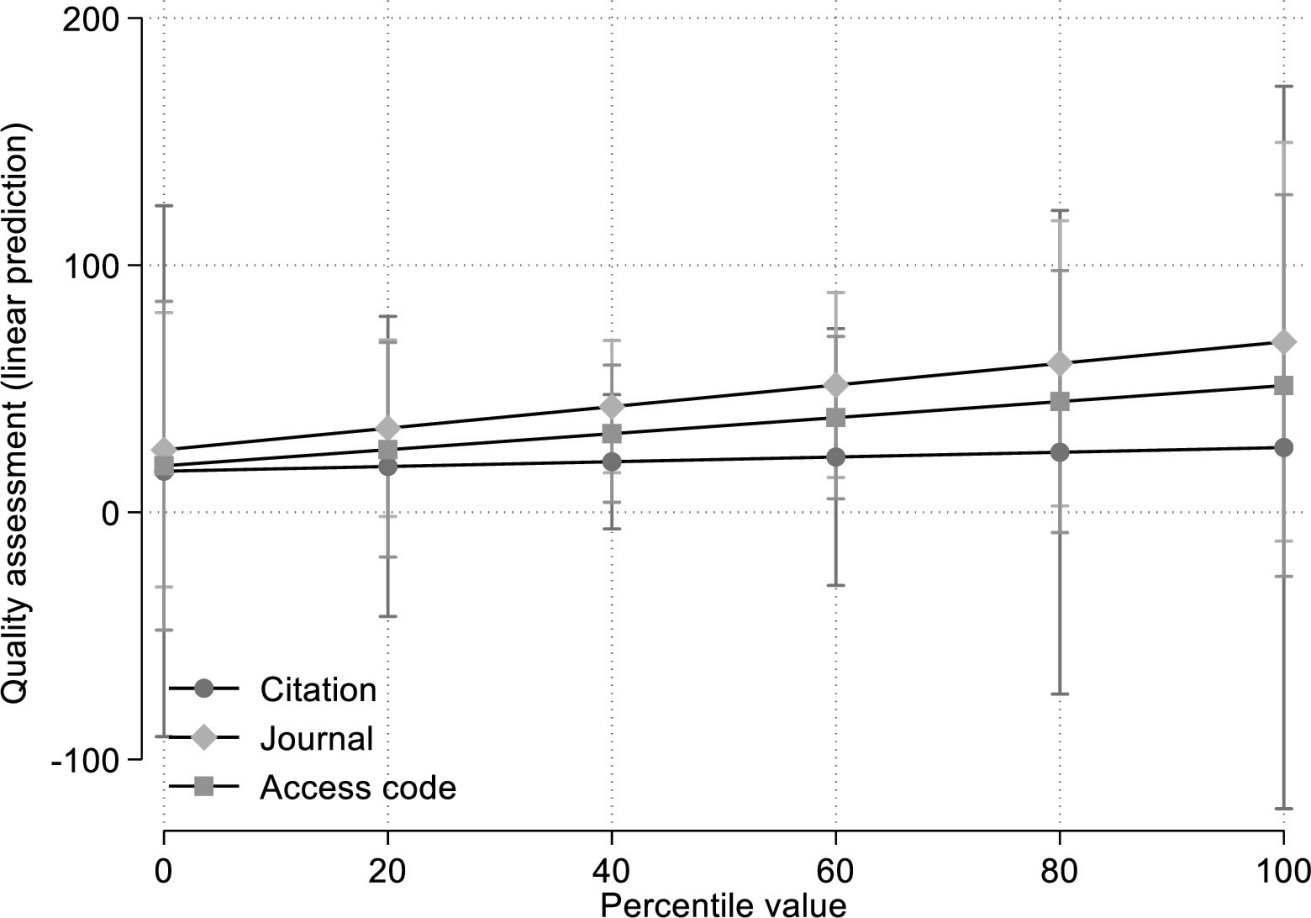
Relationships between treatments (citation, journal, and access code) and quality assessment.

In order to facilitate the interpretation of the results, this model does not include a main effect of the percentile, but three interactions. The coefficients of the interaction terms represent the effects of the percentile value on the quality assessment for the three treatments. For example, *β*_*3*_ = .095 means that an increase in the citation percentile value by 10 increases the quality assessment by .95. The predicted values from the model (which are not affected by the inclusion or non-inclusion of the main effect) are shown in Fig 2. The results show positive effects of percentile values on quality assessments for all treatments. The effect of the journal impact and access code is stronger than the effect of citations.

Data from the further questions in the web-based survey (i.e. whether the authors knew the paper, how much the reference influenced the research choices in the citing paper, and which aspects of the paper were influenced by the cited references) will be entered as possible control variables in the regression models.

We expect strong anchoring effects in the model estimations, since (1) Teplitskiy, Duede [10] reported these effects for anchors with relevant information (citation counts). (2) The literature overview by Mussweiler, Englich [11] reveal that “anchoring effects are exceptionally strong” [11].
